# Interaction between Mouse Adenovirus Type 1 and Cell Surface Heparan Sulfate Proteoglycans

**DOI:** 10.1371/journal.pone.0031454

**Published:** 2012-02-07

**Authors:** Liesbeth Lenaerts, Wim van Dam, Leentje Persoons, Lieve Naesens

**Affiliations:** Rega Institute for Medical Research, Katholieke Universiteit Leuven, Leuven, Belgium; French National Centre for Scientific Research, France

## Abstract

Application of human adenovirus type 5 (Ad5) derived vectors for cancer gene therapy has been limited by the poor cell surface expression, on some tumor cell types, of the primary Ad5 receptor, the coxsackie-adenovirus-receptor (CAR), as well as the accumulation of Ad5 in the liver following interaction with blood coagulation factor X (FX) and subsequent tethering of the FX-Ad5 complex to heparan sulfate proteoglycan (HSPG) on liver cells. As an alternative vector, mouse adenovirus type 1 (MAV-1) is particularly attractive, since this non-human adenovirus displays pronounced endothelial cell tropism and does not use CAR as a cellular attachment receptor. We here demonstrate that MAV-1 uses cell surface heparan sulfate proteoglycans (HSPGs) as primary cellular attachment receptor. Direct binding of MAV-1 to heparan sulfate-coated plates proved to be markedly more efficient compared to that of Ad5. Experiments with modified heparins revealed that the interaction of MAV-1 to HSPGs depends on their N-sulfation and, to a lesser extent, 6-O-sulfation rate. Whereas the interaction between Ad5 and HSPGs was enhanced by FX, this was not the case for MAV-1. A slot blot assay demonstrated the ability of MAV-1 to directly interact with FX, although the amount of FX complexed to MAV-1 was much lower than observed for Ad5. Analysis of the binding of MAV-1 and Ad5 to the NCI-60 panel of different human tumor cell lines revealed the preference of MAV-1 for ovarian carcinoma cells. Together, the data presented here enlarge our insight into the HSPG receptor usage of MAV-1 and support the development of an MAV-1-derived gene vector for human cancer therapy.

## Introduction

Genetically modified human adenoviruses, derived from serotype 5 (Ad5), are currently the most popular vectors used in cancer gene therapy research, the main reasons being their ability to transduce a wide range of cell types and the ease of vector propagation. However, despite encouraging early clinical advances with adenovirus vectors, their successful clinical application has been restrained because of the high seroprevalence of Ad5 in adults and the low transduction efficiency in tumor cells lacking the primary receptor for Ad5, the coxsackievirus and adenovirus receptor (CAR) (reviewed in [1]). In addition, intravenously administered Ad5 vectors predominantly accumulate in the liver, resulting in acute hepatotoxicity and dramatically diminished gene expression in target tissues [2], [3]. The hepatic sequestration of Ad5 upon intravenous administration was shown to be driven by the binding of Ad5 hexon capsid proteins to circulating blood proteins, in particular coagulation factor X (FX), which subsequently “bridge” the vector to cell surface heparan sulfate proteoglycans (HSPGs) in the liver [4], [5], [6]. HSPGs are widely distributed molecules composed of a core protein to which one or more heparan sulfate glycosaminoglycan side chains are covalently attached [7]. Due to the complex nature of the heparan sulfate biosynthesis, a wealth of different structures is produced, which enables HSPGs to bind numerous endogenous proteins with different functional properties such as growth factors, adhesion molecules and enzymes in the context of various biological processes (reviewed in [8]). HSPGs have also been recognized as initial attachment receptors of many different pathogens (for review see [9]). While it is now widely accepted that HSPGs on diverse cell types function as a receptor for Ad5 complexed to blood coagulation factors [10], [4], [11], the possibility for a direct interaction of Ad5 with HSPG is controversial. Initial research of Dechecchi and colleagues indicated that HSPGs are sufficient for Ad5 and Ad2 binding to susceptible cells [12], [13]. However, subsequent studies yielded heterogeneous findings [14], [15], [16].

To circumvent the current difficulties associated with the systemic use of Ad5 gene vectors, we focused on mouse adenovirus type 1 (MAV-1), a non-human adenovirus that could evade the restriction from pre-existing anti-Ad5 immunity in the human population and for which we previously demonstrated CAR-independent cell attachment [17]. In infected mice with advanced disease, MAV-1 is typically found in the endothelial cells of infected organs [18], [19]. A previous investigation pointed to a role for HSPGs and α_V_ integrins in the MAV-1 infection cycle, however it was not determined whether HSPGs are implicated in cellular attachment or, rather, entry of MAV-1 into susceptible cells [20]. In this study, we focused on the involvement of cell membrane HSPGs in the initial attachment of MAV-1, and examined the role of specific N- or O-sulfate groups in the HSPGs. Ad5 HSPG receptor usage was investigated in parallel. In addition, we studied the possible involvement of FX in this adenovirus-HSPG interaction. Our previous investigation revealed the ability of MAV-1 to directly associate with vitamin K-dependent coagulation factors, but opposite to Ad5, this interaction did not lead to enhanced attachment to hepatocytes [21]. We now tried to elucidate this apparent discrepancy by means of slot blot and solid phase binding assays, to analyze the binding of adenovirus-coagulation factor complexes to heparan sulfate. In addition, a profile of MAV-1 attachment to a wide range of human cancer cells was determined. Our findings increase the understanding of the initial interactions of MAV-1 with the host cell and support the interest in developing MAV-1 as an oncolytic agent.

## Results and Discussion

### MAV-1 uses HSPGs as attachment receptor

A previous investigation pointed to the importance of HSPGs in the MAV-1 infection process [20]. However, it remained to be shown at which stage of the MAV-1 infection cycle these glycosaminoglycans intervene. We therefore explored the role of HSPGs in MAV-1 attachment to susceptible cells. Since the direct HSPG interaction of Ad5, which is currently the most applied adenovirus vector, is also questioned [13], [16], [14], [15], Ad5 receptor usage was investigated as well.

**Figure 1:**
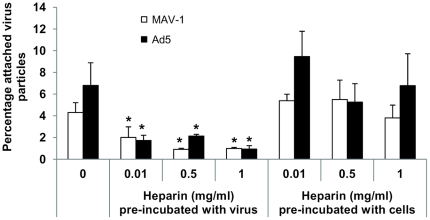
Inhibitory effect of heparin on attachment of MAV-1 and Ad5 to A549 cells. Various concentrations of heparin were pre-incubated either with cells or with virus during 1 hour at 37°C. Pretreated cells were infected with virus (3×10^3^ particles per cell) during 1 hour at 4°C. In parallel, untreated cells were chilled on ice and infected with virus (final multiplicity of infection: 3×10^3^ particles per cell) that had been pre-incubated with heparin. Subsequently, non-bound virus particles were washed away and cells were subjected to DNA-extraction for analysis by real-time PCR. Binding capacities are presented as the percentage attached virus particles relative to the total amount of added virus particles. Means (±SEM) of three independent experiments are shown (each condition performed in duplicate). *, *P*<0.05 *versus* 0 mg/ml heparin.

First, we assessed whether heparin is able to block MAV-1 or Ad5 binding to A549 cells. As a structural homolog of the highly sulfated side chains of HSPGs, heparin is often used to substitute heparan sulfate in studies of HSPG–ligand interactions. However, their composition is different since the density of N- and O-sulfation is much higher for heparin compared to heparan sulfate [22]. Pre-incubation of MAV-1 with heparin significantly inhibited MAV-1 attachment up to a maximum of 80% and inhibition was dose dependent (Fig. 1). A comparable effect was seen for Ad5 (up to 86% reduction) confirming previous investigations performed on A549 (among other) cells [13], [16]. Preloading cells with heparin did not affect MAV-1 or Ad5 attachment, showing that heparin interacts with these viruses rather than with the target cells (Fig. 1).

**Figure 2:**
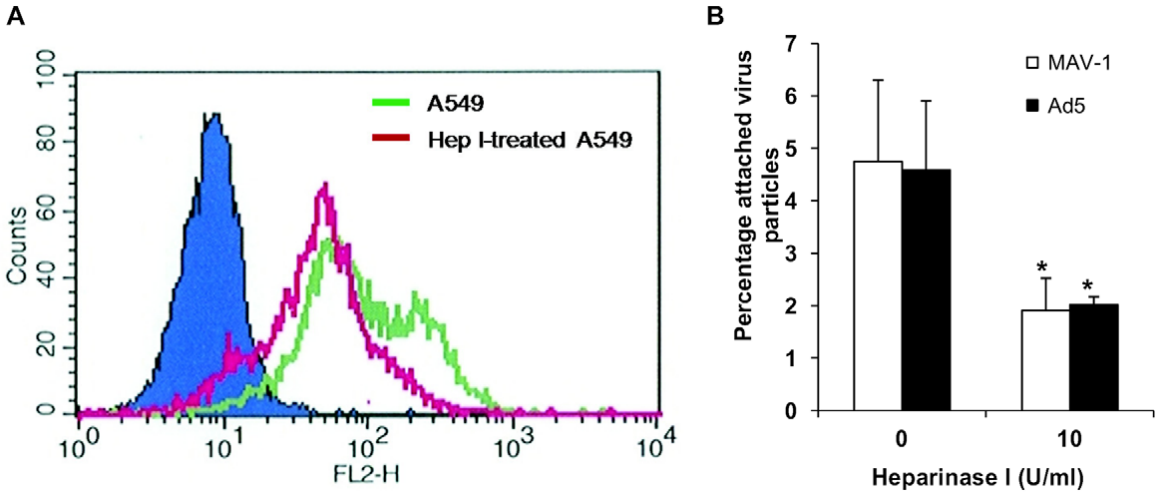
Effect of heparinase I treatment on MAV-1 and Ad5 attachment to A549 cells. **A.** FACS analysis to assess the effect of heparinase I treatment (0 or 10 U/ml during 90 min at 37°C) on heparan sulfate expression in A549 cells. Fluorescence intensity is plotted against the number of events (counts). The green line indicates untreated A549; the red line represents heparinase I-treated A549. Background in the absence of primary antibody F58-10E4 is represented by the solid blue histogram. **B.** Binding experiments in A549 cells (pretreated with heparinase I as described in A) were performed as in Fig. 1. Binding capacities are presented as the percentage attached virus particles relative to the total amount of added virus particles. Means (±SEM) of three independent experiments are shown (each condition performed in duplicate). *, P<0.05 *versus* binding in the condition 0 U/ml heparinase I.

Additional proof that MAV-1 and Ad5 can use HSPG for cellular attachment, was obtained in A549 cells pretreated with heparinase I, which cleaves heparan sulfate from the cell surface. The amount of cell surface heparan sulfate following heparinase I treatment was assessed using flow cytometry. Incubation with heparinase I led to a decline of 57% in overall cell surface heparan sulfate expression and a reduction of 84% in the population of A549 cells showing strong heparan sulfate expression (Fig. 2A). Pretreatment with heparinase I reduced MAV-1 binding to A549 cells by 60%, confirming a specific role for cellular HSPGs in MAV-1 attachment (Fig. 2B). A comparable effect was seen for Ad5 binding to A549 cells. As heparinase I treatment had no effect on cell count and cell viability (data not shown), the reduced virus attachment level could not be ascribed to toxic effects of heparinase I. Furthermore, the effect of heparinase I was specific, since a comparable treatment with chondroitinase ABC (which degrades both chondroitin sulfate and dermatan sulfate, which are the other common sulfated glycosaminoglycans present on cell membranes), had no effect on MAV-1 or Ad5 attachment to A549 cells (data not shown). This concurs with published data on the lack of effect of chondroitin sulfate A and B on MAV-1 replication [20].

**Figure 3:**
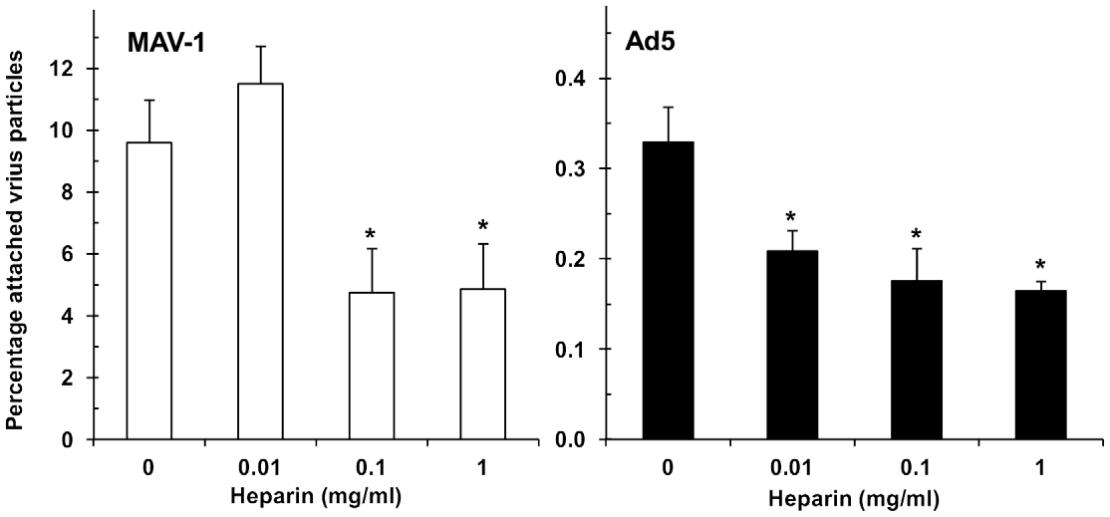
Inhibitory effect of heparin on binding of MAV-1 or Ad5 to heparan sulfate-coated plates. Adenovirus (10^10^ particles/ml in PBS) was pre-incubated with indicated concentrations of heparin for 30 min at room temperature and added to heparan sulfate-coated plates. The plates were incubated for 1 hour at room temperature and, after removal of unbound virus, attached virus particles were subjected to DNA-extraction for analysis by real-time PCR. Data are presented as the percentage attached virus particles relative to the total amount of added virus particles. Means (±SEM) of three independent experiments are shown (each condition performed in duplicate). *, *P*<0.05 *versus* 0 mg/ml heparin. Note that the Y-axis scale is different for MAV-1 (left) and Ad5 (right).

The results obtained in heparinase I-treated A549 cells indicate that HSPGs expressed on the cell surface intervene in MAV-1 and Ad5 attachment. To investigate the direct interaction of MAV-1 and Ad5 with HSPGs, we assessed whether these viruses are able to bind to heparan sulfate-coated microplates, and whether this binding is reduced in the presence of heparin. As shown in Fig. 3, the efficiency for virus binding to heparan sulfate was markedly higher for MAV-1 compared to Ad5, since the percentage of virus particles bound to the heparan sulfate-coated microplates (relative to the number of added particles) was 10% for MAV-1, but only 0.3% for Ad5. Bovine kidney heparan sulfate (which we used to coat the plates) is high in 6-O-sulfation rate, but very low in 2-O-sulfation rate [23]. It thus appears that HSPGs rich in 6-O-sulfates efficiently bind MAV-1, whereas 6-O-sulfation hampers the interaction with Ad5. When MAV-1 was preincubated with heparin, its binding to heparan sulfate was significantly inhibited. For Ad5, the already low binding to the heparan sulfate-coated plates was further (and significantly) reduced after virus preincubation with heparin.

### Binding of MAV-1 to HSPGs depends on N-sulfation and 6-O-sulfation rate

**Figure 4:**
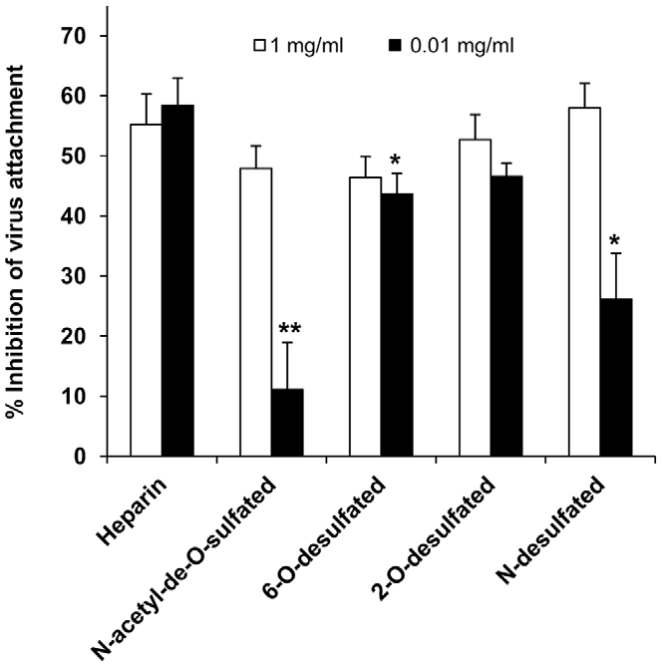
Inhibition of MAV-1 attachment to A549 cells by chemically modified heparins. A competition assay with two different concentrations of (un)modified heparin was performed essentially as described in Figure 1. Means (±SEM) of four independent experiments are shown (each condition performed in duplicate). *, *P*<0.05 and **, P<0.01 *versus* the inhibition seen with unmodified heparin.

As explained above, the efficient binding of MAV-1 to bovine kidney heparan sulfate-coated plates suggests that 6-O-sulfate groups may play a role in the MAV-1-HSPG interaction. Therefore, a cell binding competition assay with selective chemically desulfated heparins was performed. At 1 mg per ml, the desulfated heparins had similar inhibitory effect on MAV-1 binding to A549 cells as unmodified heparin (Fig. 4). However, when the compounds were added at only 0.01 mg per ml, N-desulfated heparin was significantly less effective than unmodified heparin, and a moderate (yet statistically significant) reduction in binding inhibition was also seen with the 6-O-desulfated compound. Of note, N-acetyl-de-O-sulfated heparin was hardly effective at inhibiting the binding of MAV-1 to A549 cells. In this modified heparin, all 6-O- and 2-O-sulfate groups have been removed whereas the N-sulfates have been replaced by N-acetyl functions. Taken together, these data indicate that the interaction between MAV-1 and HSPGs requires the presence of sulfate groups at the N- and, to a lesser degree, 6-O- positions.

### FX does not enhance MAV-1 attachment to endothelial cells

**Figure 5:**
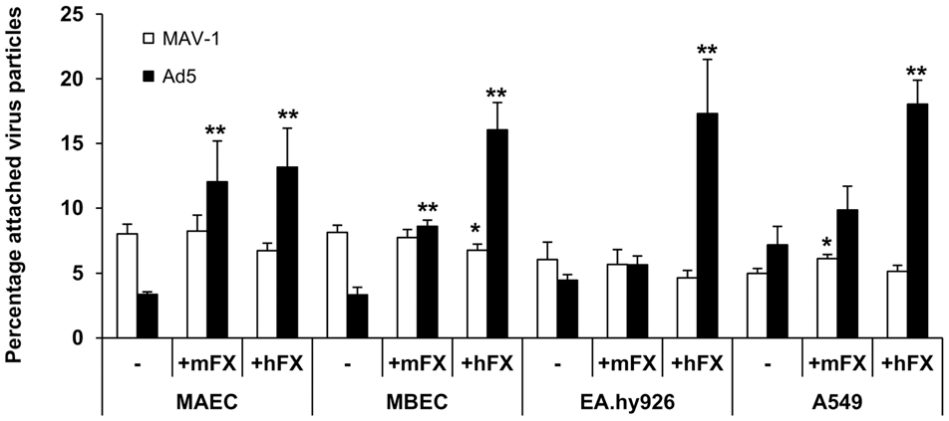
Effect of human and mouse FX zymogens on attachment of MAV-1 and Ad5 to endothelial cells. Physiologic concentrations (0.008 mg/ml) of one of the coagulation factors were pre-incubated with the endothelial cells during 45 min at 4°C, followed by incubation with virus (1×10^3^ particles per cell) for 2 hours at 4°C. Binding experiments were further performed as in Fig. 1. Binding capacities are presented as the percentage attached virus particles relative to the total amount of added virus particles. Means (±SEM) of two to three independent experiments are shown (each condition performed in duplicate). *, P<0.05 and **, P<0.01 *versus* binding in the absence of clotting factors.

Recent investigations revealed that cell surface HSPGs serve as receptors for Ad5-FX complexes, following interaction of the virus with blood coagulation factor X [24], [4]. As endothelial cells are the major target cells of MAV-1 in infected mice, the question arose as to whether FX could act as a bridging factor for MAV-1 to HSPGs on endothelial cells, or whether MAV-1 attachment to the endothelial surface is only a direct interaction. We therefore evaluated MAV-1 attachment to three different endothelial cell lines representing human macrovascular (EA.hy926), murine macrovascular (MAEC) and murine microvascular (MBEC) endothelial cells, in the presence or absence of human FX (hFX) or mouse FX (mFX). Human lung epithelial cells (A549) were included as a reference. Concurrent with previous reports describing FX-enhanced attachment of Ad5 to hepatic [4], cancer [11] or epithelial [10] cells, we found that hFX also significantly augmented Ad5 binding to the endothelial cells investigated (Fig. 5). Mouse FX only stimulated Ad5 binding to endothelial cell lines from murine origin, but not when added to human endothelial cells. A comparable phenomenon was noticed by Zaiss and colleagues who found that mFX increased adenovirus transduction in human or murine hepatic cell types, but not in non-hepatic cancer cell lines from human or mouse origin [11]. This discrepancy was ascribed to the greater affinity of the Ad-hFX complex for cellular HSPGs compared to the Ad-mFX complex. As a consequence, mFX was suggested to only increase adenovirus transduction in cells that exhibit a relatively high amount of surface HSPGs. It is therefore plausible that differences in cellular HSPG expression between human and mouse endothelial cells might also explain the lack of effect of mFX on Ad5 attachment to human endothelial cells. As shown in Figure 5, at physiologic concentrations neither mFX nor hFX affected MAV-1 attachment to any of the tested cells. These data are in agreement with our previous findings that FX does not enhance the binding of MAV-1 to hepatocytes [21], and suggest that, like for hepatic cell types, MAV-1 attachment to endothelial cells is a direct phenomenon, and putatively occurs via HSPGs.

### MAV-1 is able to bind to FX

**Figure 6:**
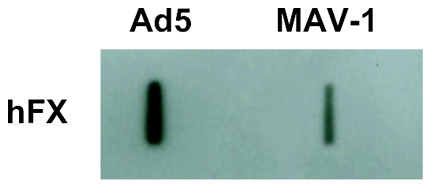
Slot blot assay demonstrating adenovirus binding to FX. Viruses (3**×**10^9^ virus particles/ml) were blotted onto PVDF membrane and subsequently incubated with hFX (0.008 mg/ml). Human FX was visualized by chemiluminescence after staining with anti-FX polyclonal antibody.

To investigate whether the observed lack of effect of FX on MAV-1 attachment resulted from the inability of MAV-1 to bind FX, we performed a slot blot analysis. Ad5 (showing strong binding to FX) was included as reference [6], [5]. Figure 6 shows that hFX binds to MAV-1 but the band was much weaker than that observed for Ad5. The ability of MAV-1 to complex with hFX confirms our previous observation that MAV-1 binds to hFX as well as to mFX immobilized on a biosensor chip [21]. Whereas the surface plasmon resonance data give insight in the binding kinetics of the MAV-1-FX interaction, the data obtained in this slot blot assay are representative for the quantity of FX complexed to the virus. The fact that the amount of FX complexed to MAV-1 is much lower than observed for Ad5, may point to structural differences in the MAV-1 hexon binding sites for FX when compared to those of Ad5. In this regard, alignment of a series of 15 human and animal adenovirus hexon proteins as performed in [25], reveals that only two out of eleven hexon amino acids (namely I421 and L426; Ad5 numbering) described to be important for FX binding [26], [5], are conserved in MAV-1 hexon.

### FX does not contribute to MAV-1 binding to heparan sulfate

**Figure 7:**
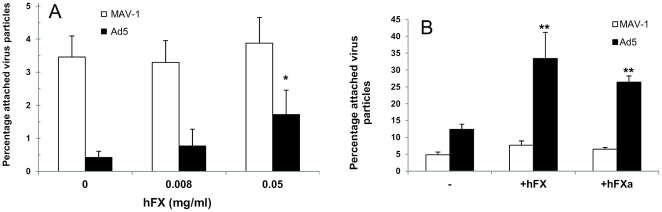
Effect of hFX or hFXa on MAV-1 or Ad5 binding. **A. Effect of hFX on virus binding to heparan sulfate-coated plates.** Adenovirus-hFX complexes were prepared by mixing virus (10^10^ particles/ml) with different concentrations of hFX. After 1 h incubation at room temperature, excess coagulation factor was removed and the virus-hFX complexes were added to heparan sulfate-coated wells for 2 hours at room temperature. Determination of the number of attached virus particles was performed as in Fig. 3. Binding capacities are presented as the percentage attached virus particles relative to the total amount of added virus particles. Means (±SEM) of three independent experiments are shown (each condition performed in duplicate). *, P<0.05 *versus* binding in the absence of FX. **B. Effect of human FX or hFXa on virus attachment to A549 cells.** Binding experiments with physiological concentrations of hFX or hFXa were performed as in Fig. 5. Binding capacities are presented as the percentage attached virus particles relative to the total amount of added virus particles. Means (±SEM) of three independent experiments are shown (each condition performed in duplicate). **, P<0.01 *versus* binding in the absence of clotting factors.

To elucidate the apparent discrepancy between the ability of MAV-1 to complex with hFX and the absence of effect of hFX in MAV-1 cell binding assays, we first investigated whether MAV-1 interaction with hFX led to the formation of a complex showing superior binding to purified HSPGs compared to non-complexed MAV-1. For Ad5, enhanced cell binding following complexation with hFX was shown to be mediated via the interaction, with cellular HSPGs, of a heparin-binding exosite (HBE) in the proteinase domain of hFX, and not through a direct interaction of the virus with the cell surface [6], [27]. We therefore allowed MAV-1 as well as Ad5 to complex with blood coagulation hFX and measured the binding of these complexes to heparan sulfate coated on a microplate. Whereas non-complexed Ad5 showed a low efficiency for binding to heparan sulfate, Ad5 binding was 2.5 and 7.5 times enhanced after complexation of Ad5 with hFX at 0.008 mg/ml (physiologic concentration) or 0.05 mg/ml, respectively (Fig. 7A). The enhancement of Ad5 binding in the presence of physiologic concentrations of hFX was in the same order of magnitude as that observed in the cell binding assay following addition of hFX (Fig. 5). In contrast to the Ad5-hFX complex, attachment of the MAV-1-hFX complex to heparan sulfate was comparable to that of virus alone, indicating that hFX does not contribute to MAV-1 attachment to heparan sulfate.

Recent investigations demonstrated that the affinity of hFX for heparin is weak but increases upon activation of the zymogen into hFXa due to conversion of the heparin-binding pro-exosite (HBPE) in the proteinase domain of hFX into the HBE of hFXa [28], [29]. We therefore wondered whether it could be possible that MAV-1 is not able to stimulate a conformational change of HBPE into HBE following complexation to hFX, which is necessary for effective heparin binding; possibly Ad5 complexation to hFX might do have this effect. To test this hypothesis, we compared hFX and hFXa (in which the HBE is exposed or contains the right conformation to bind heparin and hence HSPGs [30]) for their ability to stimulate MAV-1 binding to A549 in a cell binding assay. Ad5 was again included as a reference. hFXa significantly augmented Ad5 attachment to A549 cells to a comparable extent as hFX (Fig. 7B). This agrees with a previous observation that hFXa and its zymogen hFX had similar stimulatory effects on Ad5 transduction of HepG2 cells [4]. With regard to MAV-1, neither hFXa nor hFX significantly enhanced MAV-1 attachment to A549 cells. To ascertain that the viruses were intact after incubation with the FXa protease, MAV-1 or Ad5 samples, treated under the same conditions as applied in the A549 binding assay (i.e. 2 hours incubation at 4°C), were subjected to SDS-PAGE followed by silver staining. The band pattern was identical in buffer- and FXa-treated samples, and no signs of hexon or fiber cleavage were observed (data not shown). From the above we conclude that MAV-1 binding to FX does not lead to the formation of a complex exhibiting the right conformation for interaction with HSPGs, which might be the case for the Ad5-FX complex. However this latter hypothesis needs further investigation. Possibly, MAV-1 binding to FX may sterically hinder the accessibility of the HBE site thereby preventing subsequent FX binding to HPSG.

### Analysis of the NCI-60 panel for susceptibility to MAV-1 binding reveals a preference of MAV-1 for ovarian carcinoma cells

**Figure 8:**
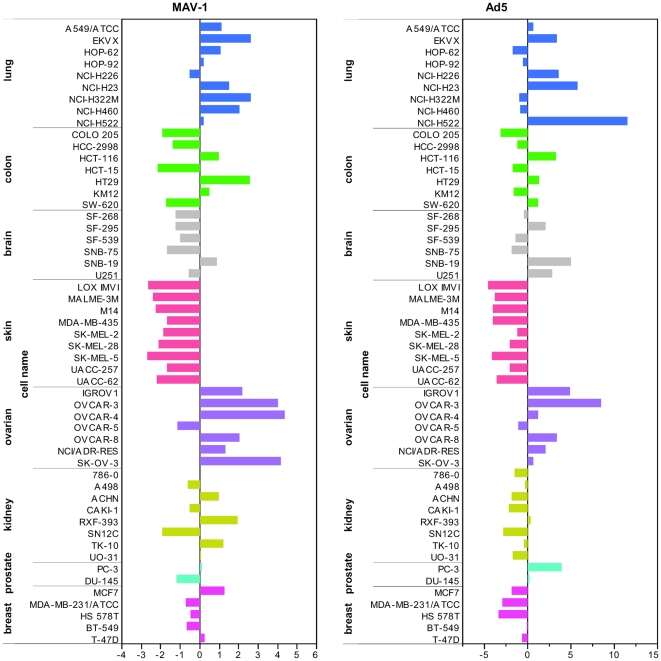
Profile of MAV-1 or Ad5 uptake by the NCI-60 tumor cell panel. Binding experiments with MAV-1 and Ad5 were essentially performed as in Fig. 1, with the exception that the incubation of virus with cells (1×10^3^ particles per cell) was performed at 37°C. The level of virus uptake is expressed relative to the mean value, averaged over all cell lines.

MAV-1 displays attractive properties for its development into a gene vector for cancer therapy [i.e. pronounced *in vivo* endothelial cell tropism, non-CAR usage, insensitivity for the FX pathway and absence of pre-existing neutralizing antibodies in human patients]. As aberrant expression of HSPGs (here validated as MAV-1 attachment receptor) is associated with tumor onset and progression in diverse tissues (reviewed in [31], [32]), we performed an extensive evaluation of MAV-1 attachment on different cancer cell types to assess its preference for certain types of cancer cells. Ad5 was included as a reference. We assessed the NCI-60 panel of human tumor cell lines for MAV-1 and Ad5 uptake and performed the experiments at 37°C in order to ensure the observation of ‘true’ viral entry, rather than the pick-up of non-specific attachment via cell surface molecules showing accidental affinity for the virus particle. All these experiments were done in the absence of any coagulation factors. The profile for attachment and internalization of Ad5 on the different tumor cell lines (Fig. 8) corresponded well with the one reported for Ad5 transduction on these cells [33], confirming the reliability of our assay. MAV-1 displayed an attachment profile different from that of Ad5, which concurs with our observations that both viruses use distinct receptors. On the whole, maximum MAV-1 uptake was seen within the set of ovarian tumor cell lines, with 2 to 5 times higher values compared to the overall mean virus uptake. These results indicate a preference of MAV-1 for ovarian carcinoma cells. In collaboration with Dr. S. Holbeck [National Cancer Institute (NCI), USA], the MAV-1 and Ad5 binding results for this NCI-60 panel were correlated to the microarray gene expression data on these cell lines, available at the NCI. More specifically, COMPARE analysis was performed to determine the Pearson correlation coefficients between our virus binding data and the gene expression microarray data for ∼10000 genes. In the case of Ad5, a clear correlation was seen between Ad5 binding efficiency and CAR mRNA expression in these NCI-60 cell lines, with a correlation coefficient of 0.57, and a P-value<0.0005. Another top correlate for Ad5 was glypican-3, a glycosylphosphatidylinositol-anchored cell membrane HSPG that regulates cell growth (either positively or negatively depending on the cell type), and is upregulated in hepatocellular and ovarian carcinoma cells [34]. This intriguing observation suggests that glypican-3 could function as an alternative receptor for CAR in Ad5 entry. For MAV-1, the list of top correlates contained one enzyme involved in HSPG biosynthesis, i.e. heparan sulfate 6-O-sulfotransferase 1 (HS6ST1). The HS6ST enzyme exists in three isoforms and catalyzes the transfer of sulfate (from adenosine 3′-phosphate, 5′-phosphosulfate) to the exocyclic C-6 of the N-sulfoglucosamine residue in heparan sulfate [35]. The COMPARE analysis yielded a correlation coefficient of 0.47 between MAV-1 binding efficiency and HS6ST1 mRNA expression, and a P-value<0.0005. The suggested role of 6-O-sulfate groups in the MAV-1-HSPG interaction concur with our binding experiments in heparan sulfate-coated plates and our binding inhibition experiments with desulfated heparins (as explained above).

In conclusion, we demonstrated that MAV-1 efficiently uses cell surface HSPGs as attachment receptor. The MAV-1-HSPG interaction requires the presence of N-sulfate and, to a lesser degree, 6-O-sulfate groups. We provide evidence that a direct (i.e. FX-independent) interaction between Ad5 and cell membrane HSPGs (such as glypican-3) is possible. Whereas the Ad5-HSPG interaction is strongly enhanced by FX, FX nor its activated form FXa add to the attachment of MAV-1 to HSPGs, possibly because the MAV-1-FX complexation may hinder subsequent FX-HSPG interactions. In the light of the demonstrated preference of MAV-1 for ovarian carcinoma cells (potentially related to their increased expression of the HS6ST1 enzyme), our data underline the potential usefulness of MAV-1-derived vectors in the oncolytic treatment of ovarian cancer.

## Materials and Methods

### Cells and viruses

The murine aortic endothelial cell line (MAEC) and the mouse brain microvascular cell line (MBEC), were a gift from Dr. M. Presta (University of Brescia, Italy). The EA.hy926 cell line, derived by the fusion of human umbilical vein endothelial cells with the continuous human lung carcinoma cell line A549 (presently the best characterized macro-vascular endothelial cell line) was purchased from the American Type Culture Collection (ATCC). All endothelial cells were maintained in Dulbecco's Minimal Essential Medium (DMEM) supplemented with 10% fetal calf serum (FCS). A549 cells (obtained from ATCC) were cultured as previously described [19]. The NCI-60 panel contains 60 well-characterized human tumor cell lines and was established at the NCI, USA. These cells were maintained in RPMI-1640 medium supplemented with 5% FCS and 2 mM L-glutamine.

Mouse adenovirus type 1 strain FL (MAV-1) was obtained from ATCC. Recombinant Ad5 (Ad5), kindly provided by Dr. A.H. Baker (University of Glasgow, United Kingdom), is an Ad5-derived recombinant virus in which the El gene region was replaced by the *lac*Z cDNA, and has been described in detail elsewhere [4]. Virus stocks were prepared in murine endothelial cells infected with MAV-1 or HEK293 cells infected with Ad5, and purified chromatographically by the Vivapure® AdenoPACK™ purification kit (Vivascience) [19]. The titers of the virus stocks, determined by real-time PCR (see below), were ∼3×10^10^ and ∼3×10^11^ viral particles per ml for MAV-1 and Ad5, respectively. All cell lines and virus stocks were free of *Mycoplasma* contamination, as determined by PCR analysis.

### Proteins and chemical compounds

Purified human FX (hFX), human FXa (hFXa) and murine FX (mFX) were obtained from Haematologic Technologies (Bio-Connect, Huissen, The Netherlands and Cambridge Bioscience, Cambridge, UK). Heparin sodium salt from porcine intestinal mucosa (MW 9–12 kDa), N-acetyl-de-O-sulfated heparin sodium salt, heparan sulfate from bovine kidney and heparinase I were purchased from Sigma-Aldrich (Bornem, Belgium). The following modified heparins were purchased from Iduron (Manchester, UK): 2-*O*-desulfated, 6-*O*-desulfated and *N*-desulfated heparin.

### Cell binding assays

One day before infection, cells were seeded into 96-well plates at 10^4^ cells per well. For competition experiments with (un)modified heparin, various concentrations of heparin were pre-incubated either with cells or with virus during 1 hour at 37°C. Cells, pre-incubated with heparin, were washed twice with ice-cold phosphate-buffered saline (PBS), chilled on ice, and infected with virus (3×10^3^ particles per cell). In parallel, untreated cells were also washed twice, chilled on ice, and infected with virus that had been pre-incubated with heparin (final multiplicity of infection: 3×10^3^ particles per cell). Following an incubation of virus with cells during 1 hour at 4°C, cells were washed extensively to remove unbound virus. Cells were subjected to cell lysis using the CellsDirect Resuspension and Lysis Buffer (Invitrogen, Merelbeke, Belgium) and the viral DNA content of the lysates was assessed by real-time PCR (see below).

For heparinase I pretreatment, cells were incubated with 75 µl heparinase I for 90 min at 37°C. Heparinase I was dissolved at 1 mg/ml in heparinase buffer (20 mM TrisHCl, 50 mM NaCl, 4 mM CaCl_2_, 0.01% BSA, pH 7.5) and further diluted to 10 U/ml in PBS supplemented with CaCl_2_ and MgCl_2_. Cells were washed twice, prechilled on ice, and incubated with virus (3×10^3^ particles per cell) during 1 hour at 4°C. Cells were washed again and subjected to cell lysis as described above.

For binding assays with coagulation factors, one of the factors was added to the cells at physiologic concentration (0.008 mg/ml), diluted in serum-free medium, and pre-incubated during 45 min on ice, followed by subsequent incubation with virus (3×10^3^ particles per cell) for 2 hours at 4°C.

Binding experiments with the NCI-60 cells were essentially performed as described above; with the exception that the incubation of virus with cells (1×10^3^ particles per cell) was performed at 37°C.

In the aforementioned experiments, control samples were included in which no compound (coagulation factor, heparin or heparinase I) was added. Uninfected controls received serum-free medium instead of virus, and were further treated in the same way as test samples. To exactly determine the total amount of virus particles added, a sample was included in which virus was not removed from the cells. Each condition was assayed in duplicate and all experiments were repeated two or three times.

### Solid-phase binding assay on heparan sulfate-coated microplates

To prepare heparan sulfate-coated plates, ELISA microtiter plates (BD™ Heparin Binding Plate, BD Biosciences, Erembodegem, Belgium) were incubated overnight at room temperature with bovine kidney heparan sulfate, diluted to 100 µg/ml in PBS. After extensive washing (three times with PBS plus 0.2% Tween-20 [PBST]) and blocking of free binding sites with 1% BSA in PBST for 1 hour at 37°C, plates were ready for use. Adenovirus (10^10^ particles/ml in PBS) was pre-incubated with indicated concentrations of heparin for 1 hour at room temperature and added to heparan sulfate-coated plates. The plates were incubated for 1 hour at room temperature and, after removal of unbound virus and extensive washing, attached virus particles were lysed using the CellsDirect Resuspension and Lysis Buffer.

Binding tests with coagulation factors were performed as described above, except for the following modifications: adenovirus-hFX complex was prepared by mixing virus (10^10^ particles/ml, in binding buffer [150 mM NaCl, 5 mM CaCl_2_, 20 mM Tris.HCl, pH 7.4]) with hFX (0, 0.008 or 0.05 mg/ml in binding buffer). Following an incubation period of 1 hour at room temperature, excess coagulation factor was removed by means of a Vivaspin ultrafiltration device (100000 MWCO; SartoriusStedim, Vilvoorde, Belgium) and the virus-hFX complex was added to the wells for 2 hours at room temperature.

Each condition was assayed in duplicate and all experiments were repeated at least three times.

### Quantification of virus particles

For determination of the number of adenovirus particles, DNA extracts were diluted ten times in RNAse-free water before subjection to SYBR® green I-based real-time PCR analysis (Platinum® SYBR® Green qPCR SuperMix-UDG with ROX; Invitrogen) in an ABI Prism 7000 apparatus (Applied Biosystems, Halle, Belgium). The specifications of this real-time PCR analysis were described previously [21].

### Slot Blot assay

Polyvinylidene fluoride (PVDF) membrane (GE Healthcare, Diegem, Belgium) was pre-wetted in methanol, rinsed with water and then with Tris Buffered Saline (TBS). A quantity of 3×10^9^ virus particles/ml in TBS was blotted onto the PVDF membrane using a Slot Blot Filtration Manifold (GE Healthcare). Following extensive washing in TBS plus 0.05% Tween-20 and incubation with TBS plus 1% milk during 30 min at room temperature, the membrane was exposed to human FX (8 µg/ml) for 1 hour at RT. Next, the membrane was incubated with rabbit anti-FX polyclonal antibody (Tebu-Bio, Boechout, Belgium; dilution 1∶200) for 60 min at RT and, subsequently, with swine anti-rabbit IgG HRP-conjugated antibody (DAKO, Heverlee, Belgium; dilution: 1∶4000) for 45 min at RT. Antibodies were diluted in TBS containing 1% milk as blocking reagent. The membrane was washed with TBS plus Tween-20 between all incubations and incubated with TBS plus 1% milk during 30 min at room temperature prior to exposure to antibodies. Antibody-bound, blotted proteins were visualized using the chemiluminescence ECL detection system (GE Healthcare).

### Flow cytometry

For FACS analysis, 3×10^5^ A549 cells were seeded in 6-well plates and 24 hours later treated with heparinase I (0 or 10 U/ml diluted in PBS supplemented with CaCl_2_ and MgCl_2_) during 90 min at 37°C. Cells were washed twice with PBS containing 2% FCS and recovered from the wells by means of a cell scraper. Then, cells were incubated with mouse anti-heparan sulfate proteoglycan antibody clone F58-10E4 (AMS Biotechnology, United Kingdom) for 30 min on ice. After washing twice with PBS containing 2% FCS, cells were stained for 30 min on ice with PE-conjugated goat anti-mouse IgG (Biolegend, Antwerpen, Belgium). Cells were washed again, fixed with 0.37% formaldehyde in PBS, and analysed on a FACScan flow cytometer with Cell Quest software (BD Biosciences). As a negative control for non-specific background staining, cells were stained in parallel with secondary antibody alone.

### Statistical analysis

Data were compared by 2-tailed Student's *t-*test for statistical significance. *P*≤0.05 was considered significant.
